# Adoption of evidence-informed guidelines in prescribing protease inhibitors for HIV-Tuberculosis co-infected patients on rifampicin and effects on HIV treatment outcomes in Uganda

**DOI:** 10.1186/s12879-021-06533-6

**Published:** 2021-08-16

**Authors:** Frank Mulindwa, Barbara Castelnuovo, Bruce Kirenga, Dennis Kalibbala, Priscilla Haguma, Martin Muddu, Fred C. Semitala

**Affiliations:** 1 Department of Internal Medicine, Makerere University College of Health Sciences, Kampala, Uganda; 2 Makerere University Infectious Diseases Institute, Kampala, Uganda; 3 Makerere University- John Hopkins University Collaboration, Kampala, Uganda; 4 Makerere University Joint AIDS Program, Kampala, Uganda

**Keywords:** Protease inhibitors, Tuberculosis, Drug-drug interactions

## Abstract

**Background:**

We aimed to determine how emerging evidence over the past decade informed how Ugandan HIV clinicians prescribed protease inhibitors (PIs) in HIV patients on rifampicin-based tuberculosis (TB) treatment and how this affected HIV treatment outcomes.

**Methods:**

We reviewed clinical records of HIV patients aged 13 years and above, treated with rifampicin-based TB treatment while on PIs between1st—January -2013 and 30th—September—2018 from twelve public HIV clinics in Uganda. Appropriate PI prescription during rifampicin-based TB treatment was defined as; prescribing doubled dose lopinavir/ritonavir- (LPV/r 800/200 mg twice daily) and inappropriate PI prescription as prescribing standard dose LPV/r or atazanavir/ritonavir (ATV/r).

**Results:**

Of the 602 patients who were on both PIs and rifampicin, 103 patients (17.1% (95% CI: 14.3–20.34)) received an appropriate PI prescription. There were no significant differences in the two-year mortality (4.8 vs. 5.7%,* P* = 0.318), loss to follow up (23.8 vs. 18.9%, *P* = 0.318) and one-year post TB treatment virologic failure rates (31.6 vs. 30.7%, *P* = 0.471) between patients that had an appropriate PI prescription and those that did not. However, more patients on double dose LPV/r had missed anti-retroviral therapy (ART) days (35.9 vs 21%, *P* = 0.001).

**Conclusion:**

We conclude that despite availability of clinical evidence, double dosing LPV/r in patients receiving rifampicin-based TB treatment is low in Uganda’s public HIV clinics but this does not seem to affect patient survival and viral suppression.

## Background

Tuberculosis (TB) remains the leading cause of HIV death, accounting for one-third of all HIV deaths worldwide [1]. Integrating HIV and TB treatment is a recommended strategy to reduce mortality and morbidity from both diseases [2, 3]. Evidence-based guidelines have been developed to optimize care for HIV-TB co-infected patients along the entire cascade from prevention [4, 5], diagnosis [6–12], timing for initiation of HIV or TB treatment [13, 14] and recognition of drug-drug interactions [15–17]. Translation of this evidence into practice has however, remained variable especially in sub-Saharan Africa where the burden of both diseases is very high [20, 21].

People living with HIV (PLHIV) develop TB at different time points, more often prior to initiation of antiretroviral therapy (ART) [22], but also, during the initial six months of ART as part of the immune reconstitution inflammatory syndrome [23, 24]. New TB disease has also been observed before initiation or during the course of protease inhibitor (PI) based second line ART [25, 26]. In this case, the management of HIV-TB co-infected patients on second line PI-based ART becomes complicated by drug-drug interactions [27], a scenario more common in resource-limited settings without rifabutin (the recommended substitute for rifampicin in patients on PIs). The World Health Organization (WHO) listed rifabutin as an essential medicine specifically to aid in the programmatic treatment of HIV-TB co-infected patients on PIs in 2009 [28]. However, because of cost, many low-middle income countries are yet to avail it in their HIV-TB programs more so in sub-Sahara African countries where the HIV-TB burden is highest [26, 29–33].

Rifampicin, a core component of treatment for drug sensitive TB induces the cytochrome P450 (CYP450) enzyme system and the efflux pump *p*-glycoprotein of which PIs are substrates. This interaction between rifampicin and PIs reduces PI trough levels by up to 90% [16, 19, 27, 34, 35]. Several pharmacokinetic studies explored how to address the inductive properties of rifampicin by increasing PI doses with variable success. Porte demonstrated that doubling the dose of lopinavir/ritonavir (LPV/r) from 400/100 mg twice a day to 800/200 mg twice a day achieved therapeutic levels of LPV when co-administered with rifampicin [27]. However, similar adjustments with other PIs including indinavir, saquinavir and atazanavir were associated with unacceptably high rates of adverse drug effects [16, 36, 37]. The above evidence informed the World Health Organization and Centre for Disease Control guidelines on the use of doubled dose LPV/r in HIV-TB co-infected patients in settings without rifabutin, with emphasis on regular clinical and laboratory monitoring of patients for toxicity [18, 38]. In Uganda, between 2013 and 2019, the recommendation for patients with TB who were receiving PI-based ART was to use rifabutin instead of rifampicin-based TB treatment and maintain the standard dose of LPV/r. For patients on atazanavir/ritonavir (ATV/r), the recommendation was to substitute ATV/r with standard dose LPV/r [39–41]. However, rifabutin was not available for use in public health facilities in Uganda during those years hence clinicians had to prescribe rifampicin based fixed dose combination TB regimens, which were routinely available.

Following the adoption of routine HIV viral load testing for monitoring HIV care in Uganda in 2015, the numbers of patients on PI based second line ART has increased [42]. This has led to an increase in the number of patients who develop TB while on PI based second line ART. There is therefore a need to optimize care in this group of patients if the third UNAIDS- 90 (viral suppression in 90% PLHIV on ART by 2020) is to be met [43]. The objective of this study was to determine if health care providers in Uganda conform to WHO guidelines regarding the prescription of PIs when treating HIV-TB co-infected patients on rifampicin-based TB therapy and whether this affects HIV treatment outcomes i.e. death, loss to follow up, missed ART doses and virologic suppression.

## Methods

### Study design, setting and participants

This was a retrospective cohort study that was conducted between December 2018 and February 2019 in twelve high volume public HIV clinics (each with at least 4500 PLHIV). All the clinics used paper-based patient treatment charts (ART cards) as the primary clinical data entry tool and an open-source electronic medical record (EMR) platform database, the Open Medical Record System (*OpenMRS*®), into which ART data was entered. HIV viral load testing was available at all the clinics, with samples processed centrally at the Uganda Ministry of Health (MoH) Central Public Health Laboratory (CPHL). Other tests such as complete blood counts, liver and kidney function tests were performed at the HIV clinics. The study clinics included nine government of Uganda owned facilities (5 Kampala City Council Authority (KCCA) clinics under the capital city management body and 4 regional referral hospital clinics) and three referral clinics that were managed by non-government organizations (Mulago Immunosuppressive syndrome (ISS) clinic, Mbarara ISS clinic and Nsambya Homecare Clinic). All study clinics had HIV and TB care integrated and ART was prescribed by medical officers (general doctors), clinical officers (diploma holding medical assistants) or nurses. For TB treatment, all the health facilities used rifampicin-based fixed dose combination anti- TB drugs during the study years.

The clinics included in this study were selected purposively from the different regions of Uganda i.e. Central, South- Western, West-Nile and Mid-Western regions. These were categorized into two groups according to the level of HIV clinical care offered: group 1 included the KCCA HIV clinics headed by general practitioners. Group 2 included Regional Referral Hospital (RRH) HIV clinics. These were also headed by general practitioners but under the referral hospital departments of medicine headed by internists. Group 2 clinics provided inpatient services, specialist clinics at the attached referral hospitals and more advanced diagnostic tests on top of what the group 1 clinics offered i.e. Outpatient ART dispensing, routine laboratory monitoring of patients on ART, HIV testing and counselling, nutritional counselling and care and TB care.

We consecutively included HIV patients ≥ 13 years diagnosed and treated for TB while on PI- based second line ART between 1st-January-2013 and 30th—September- 2018. A list of eligible patients was obtained from Open MRS. Patients whose paper ART cards were missing and those that were treated for TB for > 1 year were excluded for possible multi-drug resistant TB treatment.

### Definitions

We defined standard dose LPV/r as LPV/r 400/100 mg taken twice daily (BID), dose adjusted LPV/r as 800/200 mg (BID). Appropriate PI prescription during rifampicin-based TB treatment was defined as prescribing LPV/r 800/200 mg twice daily. Inappropriate PI prescription was defined as prescribing standard dose LPV/r or ATV/r during rifampicin- based TB treatment [18] 38.

Virologic failure was defined as a documented HIV viral load ≥ 1000 copies /ml within a year post TB treatment [39–41]. Death was defined as documented death as reported in the ART register. Loss to follow up was defined as; no clinical visit for ≥ 3 months after missing a scheduled appointment or pharmacy pick-up visit [39–41]**.**

### Data collection

We extracted data from the patients’ HIV treatment charts where a TB treatment code 3 or 4 was used to indicate that the patient was on TB treatment during particular clinic visits. This code however did not specify the TB site. Data extracted included; sex, age, weight and functional status at start of TB treatment, duration on PI-based ART before start of TB treatment, ART adherence during TB treatment (measured by pill counts), number of liver function tests done during HIV-TB treatment and the cadre of health worker who prescribed ART at the start of TB treatment. The outcome measurements collected included: the PI prescribed and whether or not dose adjustment was made for LPV/r-based ART at the start of TB treatment, viral load results within a year post TB treatment completion, missed ART doses during TB treatment and two-year post TB treatment initiation date follow up status (alive in care, lost to follow up, dead or transferred out).

All data collected from the electronic medical record were double checked for accuracy using the hard copy treatment charts which were the primary data collection tools during routine clinical care.

### Statistical analysis

Continuous data were summarized as medians with interquartile ranges, while categorical data was summarized as frequencies and percentages. Chi-square test was used to compare baseline clinical and demographic characteristics of patients who had an appropriate PI prescription while on rifampicin-based TB treatment and those that did not.

The overall proportion of patients with appropriate PI prescriptions while on rifampicin-based TB treatment was calculated. It was also represented on a trend line graph for each year from 2013 to 2018 by clinic type. Bivariate and multivariate logistic regression was performed to determine factors associated with inappropriate PI prescription with the following variables: cadre of health care workers prescribing ART (doctor, nurse, clinical officer), clinic cluster, age (stratified in < 25, and then in 10 years increment), weight (stratified by weight brackets used to determine the number of fixed dose combination anti-TB pills) and duration on protease inhibitor before TB treatment was initiated treatment. Variables with a p value ≤ 0.2 in the adjusted analysis were included in the final model.

We also used chi- square test to compare the proportions of HIV treatment outcomes in both patient groups. The analysis was done using STATA version 14.0 (STATA Corp., Texas, USA).

## Results

**Fig. 1:**
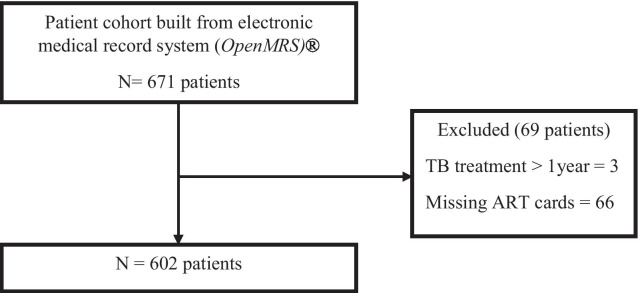
Study enrollment schema for study patients

We evaluated electronic medical records of 671 PLHIV who were concurrently treated with protease inhibitors and anti-TB therapy. Of these, 69 patients (10.2%) were excluded from the study (3 patients were on TB treatment for longer than one year, while 66 patients had their ART treatment charts missing). Of the remaining 602 patients, 248 (41.2%) had a two year follow up status at the time of data collection (had been treated for TB between 1st—January 2013 and 28th—February 2017) and 218 (36.2%) had a documented viral load result within a year of completion of TB treatment (Fig. 1).

### Baseline demographic and clinical characteristics

More than half of the patients 314, (52.16%)) were male, with a majority (498, 82.72%) from Regional Referral Hospital HIV clinics and 104 patients (17.28%) from KCCA clinics. The median age (IQR) was 35 [16, 26–42] years. The median (IQR) time the patients were on a PI-based therapy, before initiation of TB treatment, was 7.1 (0–24.6) months. Nearly a third of the patients (185, 30.73%) started the PI-based ART regimens on the same day as for the TB treatment. Half of the patients, 303 (50.33%) weighed 40–54 kg and therefore qualified for 3 fixed dose combination TB pills per day. This was followed by the 55–70 kg weight range (198, 32.89%) corresponding to 4 fixed dose combination TB pills per day as recommended by the Uganda Ministry of Health [44]. 71 patients (11.79%) had at least one liver function test done during TB therapy and 459 (76.25%) patients had ≥ 95% reported ART adherence during TB therapy (Table 1).

**Table 1:** Overall and chi-square test comparative demographic and clinical characteristics of the study patients

Characteristics	Overall cohort (n = 602)	Appropriate PI prescription (n = 103)	Inappropriate PI prescription (n = 499)	*p*-value
Gender n (%)				
Female	288 (47.8)	43 (41.7)	245 (49.1)	0.174
Clinic level n (%)				0.004
Kampala City Council HIV Clinics	104 (17.3)	2 8 (26.9)	76 (73.1)	
Regional Referral HIV clinics	498 (82.7)	75 (15.1)	423 (84.9)	
Age at start of TB treatment (years), median (IQR)	35 (26–4)			0.033
< 25, n (%)	131 (21.8)	22 (16.8)	109 (83.2)	
25–34, n (%)	165 (27.4)	31 (18.8)	134 (81.2)	
35–44, n (%)	179 (29.7)	29 (16.2)	150 (83.8)	
45–54, n (%)	100 (16.6)	12 (12.0)	88 (80.0)	
55–64, n (%)	22 (3.7)	9 (40.9)	13 (59.1)	
> 64, n (%)	5 (0.8)	0	5 (100.0)	
Number of months on PI before TB therapy, median (IQR)	7.15 (0.0–24.6)			0.004
PI and TB treatment started on the same date, n (%)	185 (30.7)	45 (43.7)	140 (28.1)	
0–6 months, n (%)	106 (17.6)	22 (21.4)	84 (16.8)	
7–12 months, n (%)	56 (9.30)	9 (8.7)	47 (9.4)	
13–24 months, n (%)	102 (16.9)	12 (11.7)	90 (18.0)	
> 24 months, n (%)	153 (25.4)	15 (14.6)	138 (27.7)	
Weight at start of TB treatment (kg) n (%)				0.022
< 33	30 (5.0)	8 (26.7)	22 (73.3)	
33–39	44 (7.3)	9 (20.5)	35 (79.6)	
40–54	303 (50.3)	44 (14.5)	259 (85.5)	
55–70	198 (32.9)	32 (16.2)	16 (83.8)	
> 70	27 (4.5)	10 (37.0)	17 (63.0)	
Functional status at start of TB treatment n (%)				
Ambulatory	91 (15.1)	14 (15.4)	77 (84.6)	0.604
Bedridden	27 (4.5)	4 (14.8)	23 (85.2)	
Working	484 (80.4)	85 (17.4)	399 (82.6)	
Number of LFTs done during PI-TB treatment n (%)				
0	531 (88.2)	99 (18.6)	432 (81.4)	0.006
≥ 1	71 (11.8)	4 (5.6)	67 (94.4)	
Reported ART adherence during TB treatment n (%)				
≥ 95%	459 (76.3)	79 (17.2)	380 (82.8)	0.805
85–94%	50 (8.3)	7 (14.0)	43 (86.0)	
< 85%	93 (15.5)	17 (18.3)	76 (81.7)	

*Appropriate PI prescription * prescription of LPV/r 800/ 200 mg BID, *inappropriate PI prescription* prescription of LPV/r 400/100 mg BID or a prescription of ATV/r, *ART* Anti-retroviral therapy, *LFTs * Liver function tests, *IQR* Inter-quartile range

### PI prescription during rifampicin-based TB treatment

Of the 602 patients who were taking both PIs and rifampicin-based TB therapy, 103 (17.1% (95% CI: 14.3–20.34)) had an appropriate PI prescription. Of the 499 patients that had inappropriate PI prescriptions, 223 patients (37% (95% CI: 33.26–40.99)) had a prescription of ATV/r and 276 (45.9% (95% CI: 41.89–49.86)) a prescription of standard dose LPV/r. Doctors and nurses were less likely to prescribe protease inhibitors inappropriately (OR: 0.26, 95% CI: 0.15–0 0.46, OR: 0.35, 95% CI: 0.18–0 0.71 respectively, *P* < 0.001) as compared to clinical officers. However, there was no difference in prescription by clinic level, patients’ age or weight, and duration on PI (Table 2).

**Table 2:** Bivariate and multivariate logistic regression for factors associated with inappropriate PI prescription during rifampicin-based TB treatment

	Crude Odds ratio (95% CI)	*P* value	Adjusted Odds ratio (95% CI)	*P* value
Cadre of prescribing clinician				
Clinical officer	1		1	
Doctor	0.22 (0.13–0.36)	< 0.001	0.26 (0.15–0 .46)	< 0.001
Nurse	0.27 (0.15–0.47)	< 0.001	0.35 (0.18–0 .71)	0.003
Clinic level				
KCCA	1		1	
RRH	2.08 (1.26–4.18)	0.004	1.10 (0.60–2.03)	0.760
Age (years)				
< 25	1		1	
25–34	0.87 (0.47–1.59)	0.657	0.86 (0.42–1.76)	0.679
35–44	1.04 (0.56–1.91)	0.889	0.97 (0.47–2.01)	0.939
45–54	1.48 (0.69–3.16)	0.310	1.24 (0.52–2.96)	0.629
55–64	0.29 (0.11–0.76)	0.012	0.33 (0.11–1.02)	0.053
Weight (kg)				
< 33	1		1	
33–39	1.41 (0.47–4.21)	0.534	1.10 (0.33–3.70)	0.878
40–54	2.14 (0.89–5.10)	0.086	1.95 (0.71–5.38)	0.197
55–70	1.88 (0.77–4.61)	0.164	1.78 (0.59–5.32)	0.305
> 70	0.62 (0.20–1.90)	0.402	0.49 (0.12–1.94)	0.310
Duration on PI before TB treatment				
PI and TB treatment started the same day	1		1	
< 1 year	1.35 (0.81–2.27)	0.245	1.01 (0.58–1.77)	0.975
1 year–≤ 2 years	2.41 (1.21–4.80)	0.012	1.45 (0.69–3.05)	0.322
> 2 years	2.96 (1.57–5.55)	0.001	1.56 (0.77–3.16)	0.216

*KCCA* Kampala City Council Authority, *RRH* Regional Referral Hospital, *inappropriate PI prescription * prescription of LPV/r 400/100 mg BID or a prescription of ATV/r

### HIV treatment outcomes

More PLHIV in the appropriate PI prescription group had missed ART days determined by pill counts as compared to those who received inappropriate PI prescriptions (35.9 vs. 21%, *P* = 0.001). There were no significant differences in the two-year follow up outcomes i.e. mortality and loss to follow up between patients that received double dose LPV/r and those that did not (4.8 vs. 5.7%, 23.8 vs. 18.9%, *P* = 0.318 respectively). Of the 218 patients with a viral load done within 1-year post TB treatment end date and results documented, there was no significant difference in the rates of virologic failure in the appropriate PI prescription group and inappropriate PI prescription group (31.6 vs. 30.7%, *P* = 0.471) (Table 3).

**Table 3:** Chi square comparative HIV treatment outcomes among the study patients by PI prescription

	Appropriate PI prescription n (%)	Inappropriate PI prescription n (%)	*p*-value
2-year post TB treatment HIV clinical outcome (N = 248)	N = 21	N = 227	0.318
Alive in care	13 (61.9)	146 (64.3)	
Dead	1 (4.8)	13 (5.7)	
LTFU	5 (23.8)	43 (18.9)	
TO	2 (9.5)	25 (11.0)	
Missed ART days during TB treatment, (N = 602)	N = 103	N = 499	0.001
None	66 (64.1)	394 (79.0)	
Missed	37 (35.9)	105 (21.0)	
Closest viral load post TB treatment (within 1 year) ( N = 218)	N = 19	N = 199	0.471
Suppressed	13 (68.4)	138 (69.4)	
Virologic failure	6 (31.6)	61 (30.7)	

*LTFU *loss to follow up*, TO *Transferred out*, Appropriate PI *prescription LPV/r 800/ 200 mg BID*, inappropriate PI *prescription prescription of LPV/r 400/100 mg BID or a prescription of ATV/r*, ART *Antiretroviral therapy

## Discussion

**Fig. 2:**
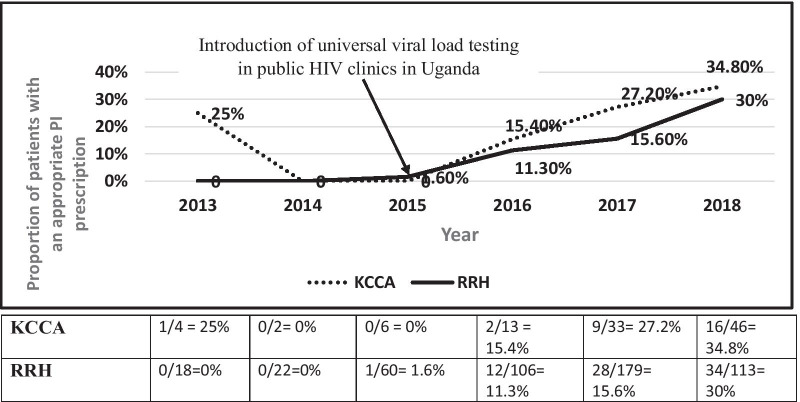
Trends of proportion of patients with appropriate PI prescription by clinic level and year. *KCCA* Kampala Capital City Authority, *RRH* Regional Referral Hospital

In this retrospective cohort study, we assessed how emerging evidence over the past decade informed Ugandan HIV clinicians’ prescription practices for PIs when treating HIV-TB co-infected patients on rifampicin-based TB treatment. We found that less than a fifth (17.1%) of eligible patients had an appropriate PI prescription during rifampicin-based TB therapy. This may be attributed to the absence of clear national HIV-TB treatment guidelines on using dose adjusted LPV/r as an option in the absence of rifabutin for TB treatment [39–41]. Additionally, rifabutin is not provided by the Uganda Ministry of Health TB program and hence not routinely available in public clinics [44]. These observations are currently very relevant given the increasing number of patients on PI- based ART and TB treatment since the introduction of routine universal viral load monitoring in 2015 (Fig. 2).

In this study, we also found that doctors were more likely to appropriately prescribe PIs as recommended, compared to clinical officers. This is expected since doctors receive more clinical training than clinical officers. Not surprising, nurses also prescribed PIs more appropriately as compared to clinical officers. This may be attributed to the fact that, there has been deliberate task shifting drives in Ugandan HIV clinics to empower nurses to prescribe ART [45, 46]. We however, did not find association between the clinic level and PI prescription despite Regional Referral Hospital HIV clinics being at a higher health care delivery level than the KCCA clinics. This can be attributed to system strengthening efforts offered to KCCA clinics by the President’s Emergency Plan for AIDS Relief (PEPFAR) program through the Infectious Diseases Institute (IDI), one of the implementation partners in Uganda.

This study further found no differences in two-year follow up status and virologic failure rates between patients that had appropriate PI prescriptions and those that did not. We did however find equally high levels of virologic failure in both groups, 31.6% and 30.7%. This is high compared to a retrospective multicenter Sub-Saharan study of which one site was in Kampala- Uganda, that demonstrated 8% two year virologic failure rates in a PI based ART cohort [47]. The same study demonstrated rifampicin exposure as a risk factor for virologic failure underlying the fact that patients on second line PI based ART co-infected with TB are at a higher risk of treatment failure. It is possible that some of the patients in our analysis developed TB in the first place because of treatment failure while on PIs as was demonstrated in a South African cohort study that showed a high viral load as an independent risk factor for development of TB [48], or the viremia was transient due to the TB infection [49, 50]. Additionally, it is also possible the 218 (36.2%) patients that got a viral load done within a year post TB treatment are the patients who clinically showed symptoms of treatment failure and hence were prioritized. These reasons could partially account for the markedly high rates of virologic failure in the study group.

Comparatively, a retrospective study in South Africa that compared tolerability and virologic outcomes between patients who were treated with dose-adjusted LPV/r to those on standard dose LPV/r while on rifampicin also found no difference in virologic suppression between the two groups post TB therapy. One shortcoming of this study was the small sample size of 29 patients [17]. The same study also demonstrated a high proportion of patients developing gastrointestinal and hepatic toxicity in the group that received double dosed LPV/r with an increased need for treatment discontinuation (47% vs. 7%; p = 0.035). Another pharmacokinetic study in Brazil that evaluated the interaction between LPV/r 800/200 mg BID and rifampicin demonstrated that most patients attained therapeutic levels of LPV but notably and clinically very important, adherence to both TB treatment and PIs was low because of the high pill burden and increased frequency of adverse events [19]. In our study, patients who received appropriate PI prescriptions had more missed ART days as compared to those that did not, suggesting that adherence may have been poorer in this group most likely due to the high pill burden and side effects associated with LPV/r dose adjustment. This finding also suggests that while the drug levels are potentially within the therapeutic range in patients with appropriate PI prescriptions, this may be negated by suboptimal adherence [19].

From the study results, we recommend a deliberate training program for HIV health care providers in Uganda with more emphasis on clinical officers on the need for dose adjusting LPV/r among patients on PI-based ART while taking rifampicin- based TB treatment. There should as well be more robust clinical and laboratory follow up in these patients to improve drug adherence as well as effectively monitor for adverse events.

Limitations of this study included the retrospective nature of the analysis and the use of observational data as well as not measuring TB outcomes of the patients. We only had post TB treatment viral load results of a third of the patients which may not be representative of the whole study population. Additionally, we were also not able to compare post TB treatment viral loads with pre-TB treatment viral loads since most patients did not have viral loads done prior or at the start of TB treatment. Our study had multiple strengths including the fact that it was multi-center and included different regions of the country and therefore likely to be representative. Also, the proportion of missing patient files was acceptable at 9.8% (< 10%).

## Conclusion

Appropriate PI-based ART prescription in patients receiving rifampicin-based TB treatment is low in Uganda’s public HIV clinics. This however, did not seem to influence the patient follow up status and virologic suppression.

## Data Availability

The datasets used and/or analyzed during the current study are available from the corresponding author on reasonable request.

## Abbreviations

ART: Anti-Retroviral Therapy
ATV/r: Atazanavir/ritonavir
CYP450: Cytochrome P450
CDC: Center for Disease Control
CPHL: Central Public Health Laboratory
EMR: Electronic Medical Record system
ISS: Immunosuppressive syndrome
IDI: Infectious Diseases Institute
KCCA: Kampala City Council Authority
LPV/r: Lopinavir/ ritonavir
MoH: Ministry of Health
PEPFAR: Presidential Emergency Fund for AIDS Relief
PIs: Protease Inhibitors
PLHIV: People Living with HIV
RRH: Regional Referral Hospital
TB: Tuberculosis
